# Development of transomental hernia shortly after laparoscopic colonic surgery: a case report

**DOI:** 10.1186/s40792-020-0783-6

**Published:** 2020-01-08

**Authors:** Takahiro Haruna, Akihisa Matsuda, Michihiro Koizumi, Takeshi Yamada, Seiichi Shinji, Yasuyuki Yokoyama, Goro Takahashi, Masahiro Hotta, Takuma Iwai, Keisuke Hara, Kohki Takeda, Hiroshi Yoshida

**Affiliations:** Department of Gastrointestinal and Hepato-Biliary-Pancreatic Surgery, Nippon Medical School, 1-1-5 Sendagi, Bunkyo, Tokyo, 113-8603 Japan

**Keywords:** Transomental hernia, Postoperative internal hernia, Laparoscopic colorectal surgery

## Abstract

**Background:**

A transomental hernia is defined as bowel invagination into an abnormal hiatus of the omentum. It is a rare type of internal hernia that is sometimes lethal. We herein report a case of a transomental hernia developing shortly after laparoscopic sigmoidectomy.

**Case presentation:**

A 71-year-old man underwent laparoscopic sigmoidectomy. He was admitted to our hospital because of abdominal pain and nausea on postoperative day 12. Laboratory investigation showed increased levels of inflammatory markers. Abdominal computed tomography showed a closed loop and mesenteric edema of the small intestine with ascites. We performed an emergency operation under the diagnosis of strangulated bowel obstruction. Operative findings showed internal herniation of strangulated ileal loops through a defect of the omentum with hemorrhagic ascites. The incarcerated small bowel was resected and reconstructed because the ischemic change was irreversible after the reduction. We partially resected the omentum that had formed the defect. The patient’s postoperative progress was good, and he was discharged on postoperative day 8.

**Conclusions:**

Almost all internal hernias after intestinal surgery are mesenteric hernias; however, we should bear in mind that the more lethal transomental hernia is also possible. Therefore, immediate surgical exploration should be performed in a timely manner for internal hernias, especially for patients with early-onset symptoms after laparoscopic intestinal surgery.

## Background

An internal hernia is an acute or chronic protrusion of the small bowel through a mesenteric or peritoneal orifice. Hernias account for 5.8% of small bowel obstructions [1]. A transomental hernia is defined as bowel invagination into an abnormal hiatus of the omentum. This type of hernia constitutes only 1% to 4% of all internal hernias and is occasionally lethal because of bowel strangulation [2]. Most transomental hernias are congenital; however, they are also rarely traumatic or iatrogenic [3, 4]. We herein report the first case of a transomental hernia that developed shortly after laparoscopic sigmoidectomy.

## Case presentation

A 71-year-old obese man (body mass index, 25.1) was admitted to Nippon Medical School Hospital for the treatment of sigmoid colon cancer (cT3, N0, stage IIa: JSCCR classification, ninth edition). He had no history of abdominal surgery. He underwent multi-port conventional laparoscopic sigmoidectomy with a transection of the root of inferior mesenteric artery (D3). Most dissecting procedures were performed with monopolar electrocautery. Regardless of abundant visceral fat, omentum was relatively thin and partially transparent without any adhesion to the abdominal wall and adjacent organs. No obvious omental damage was observed during the mobilization and the take-down of splenic flexure was not required for tension-free anastomosis using a double stapling technique. Mesocolic defect was not repaired; however, any omental hiatus was not visualized at the end of the surgery. The operation time was 221 min and the estimated blood loss was 7 ml. His postoperative course was uneventful, and he was discharged on postoperative day 8 without any abnormality in laboratory data.

**Fig. 1:**
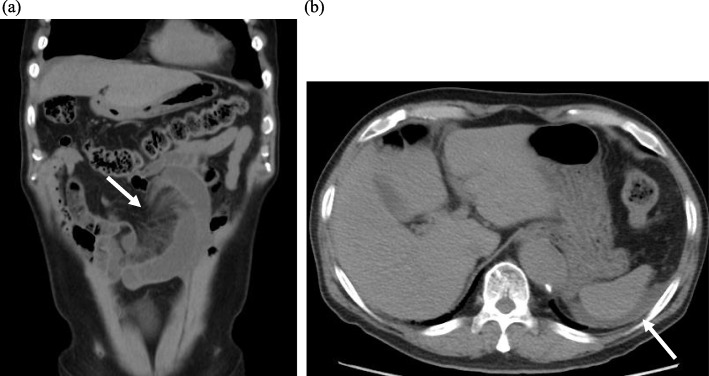
Computed tomography findings. **a** Computed tomography revealed centralization of the mesenteric vessels and turbidity of the small intestine mesenteric fat around these vessels (arrow). These findings indicated incarceration and ischemic change of the small intestine. **b** The presence of fluid with high density under the diaphragm (arrow) indicated bloody ascites

He visited our emergency outpatient unit because of abdominal pain and nausea on postoperative day 12. His vital signs were not abnormal, such as 36.7 °C of body temperature, 159/80 mm/Hg of blood pressure, 77 beats per minute of heart rate, 18 breath per minute of respiratory rate, and 99% of oxygen saturation with pulse oximetry at room air. Physical examination revealed a distended abdomen with periumbilical pain without rebound tenderness. Laboratory investigation showed an increased white blood cell count (14,900/mm^3^) and LDH (247 U/L) and normal levels of C-reactive protein (0.03 mg/dl) and creatinine (1.05 mg/dl). A simple abdominal computed tomography (CT) showed a closed loop and mesenteric edema of the small intestine with ascites, which strongly indicated strangulated bowel obstruction (Fig. 1). We decided to move to an immediate surgery based on this CT and physical findings without contrast enhancement.

**Fig. 2:**
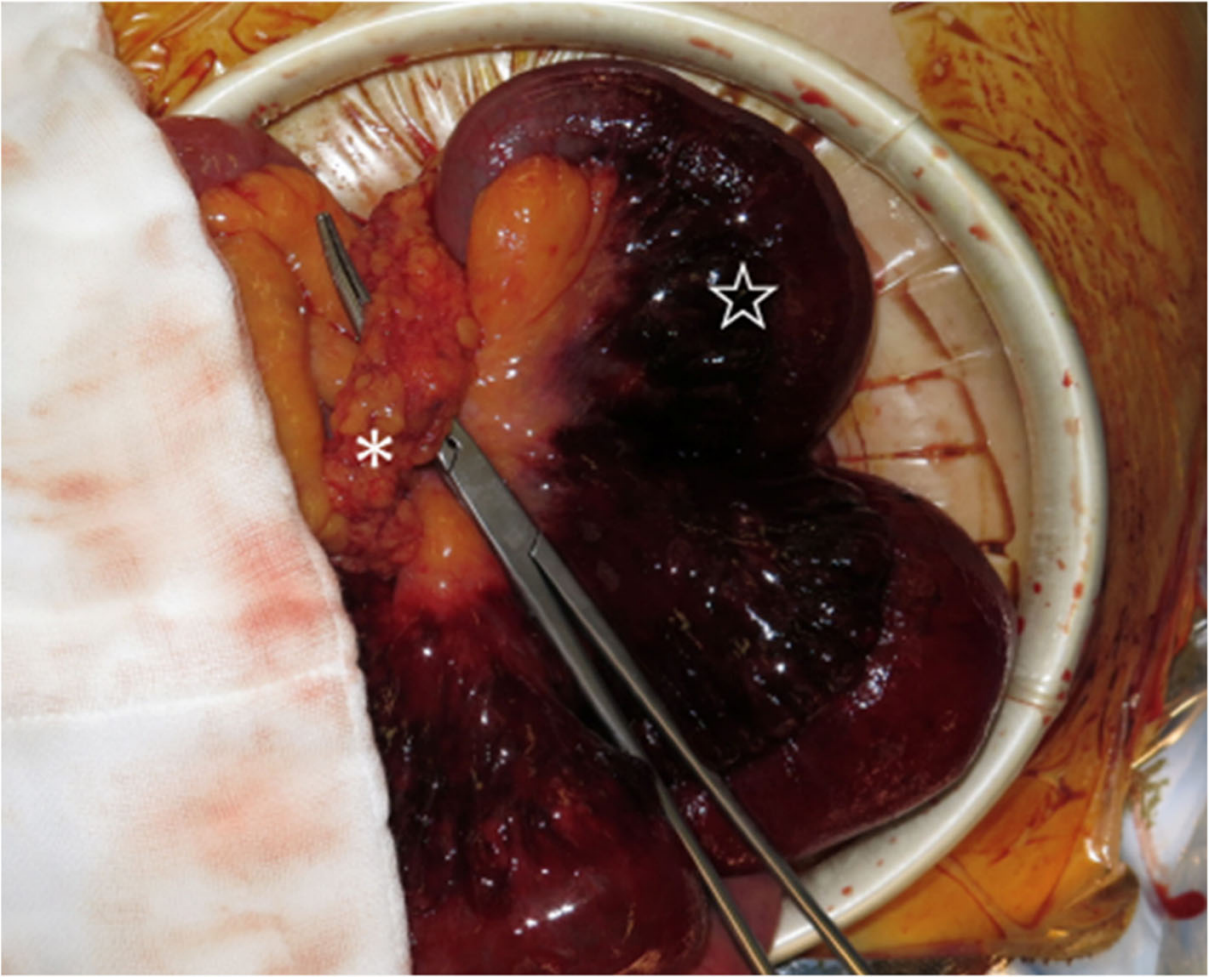
Intraoperative findings. The small intestine was incarcerated through the omental hiatus (※) from the right to left side. The intestine (☆) exhibited necrotic change

An emergency operation with a midline incision revealed internal herniation of nonviable small bowel through an omental defect with hemorrhagic ascites (Fig. 2). The omental defect was approximately 3 cm in diameter. The incarcerated small bowel was released by dividing the omentum. A 50-cm-long section of incarcerated small bowel approximately 150 cm proximal to the ileocolic junction was resected and reconstructed with functional end-to-end anastomosis because the ischemic change was irreversible after the reduction. We resected the part of the omentum that had formed the defect. The patient’s postoperative progress was good, white blood cell count and LDH improved gradually after the surgery, and they were normal levels on postoperative day 3. He was discharged on postoperative day 8. At the 17-month follow-up consultation, the patient had no complaints, and his intestinal transit had been re-established.

## Discussion

We experienced a case involving a transomental hernia that developed shortly after laparoscopic sigmoidectomy. A transomental hernia is a rare complication of surgery, and this is the first report of a transomental hernia that developed after laparoscopic colorectal surgery.

Transomental hernias are particularly rare, constituting only 1% to 4% of all internal hernias; only 24 cases have been reported in the English-language literature [5]. The pathogenesis of a transomental hernia involves the development of a congenital defect of the omentum and omental fragility caused by emaciation or atrophy of the omentum secondary to aging, oral steroids, trauma, surgery, or inflammation [3, 4]. In the present case, no congenital omental defect was found, and the omentum was not dissected or roughly manipulated during the initial laparoscopic surgery. Additionally, factors including (1) omentum was relatively thin and transparent partially, and (2) no obvious omental adhesions to abdominal wall and adjunct organs were observed at the re-laparotomy, led us to make a conclusion that initial surgery had no direct causal effect with the occurrence of transomental hernia, however, could have influenced the secondary development of ometal fragility, followed by the formation of a hernial defect.

The short-term benefits and safety of laparoscopic surgery for colorectal cancer have already been demonstrated; however, a certain number of patients could develop postoperative complications [6, 7]. Although the incidence of an internal hernia after laparoscopic colorectal surgery is low (0.65%) [8], most internal hernias are caused by a previous surgical procedure [1]. Left-sided colonic resection accounts the vast majority of primary laparoscopic surgeries that result in internal hernias. Most internal hernias are transmesocolic [8]. One plausible cause is the formation of a relatively large defect of the mesocolon secondary to the procedure. Most such large mesocolic defects are not easily repaired because of the technical difficulty. Postoperative internal hernias frequently occur 4 months after surgery [8]. In contrast, the internal hernia in the present case occurred just 12 days after surgery. The reason for the short time to onset in our case is unknown; however, two hypotheses are reduction of intra-abdominal adhesions with laparoscopic surgery and increased intra-abdominal pressure with early mobilization as part of an enhanced recovery program.

Seven types of internal hernias have been described: paraduodenal, pericecal, foramen of Winslow, intersigmoid, transmesenteric, transomental, and retroanastomotic hernias. Transomental hernias are the rarest type [9]. Yamaguchi [10] classified transomental hernias as type A (peritoneal cavity → greater omentum → peritoneal cavity), B (peritoneal cavity → omental bursa → peritoneal cavity), or C (peritoneal cavity → omental bursa). Type A is the most the common, and the hernia in the present case was also type A.

In general, the preoperative diagnosis of an internal hernia itself is not challenging [5]. Characteristic CT findings and the patient’s history allowed us to diagnose the internal hernia in this case; however, we could not identify the specific pathogenesis. Despite the high mortality of transomental hernias (30%) [2, 3], the definitive diagnosis is usually established intraoperatively, as in our case [11]. Ito et al. [12] recently reported the clinical utility of the CT finding of displacement of the transverse colon posterior to obstructed intestinal loops, which distinguishes transomental hernia from other types of small bowel obstruction.

In this case, we resected the part of the omentum that formed the defect to eliminate the potential for future bowel incarceration. Korn et al. [13] reported complete omental resection in a patient with Swiss cheese omentum containing multiple defects of varying size. In contrast, Cao et al. [14] reported the closure of multiple mesenteric defects to preserve the omentum and thus prevent adhesional bowel obstruction.

A transomental hernia is a rare cause of small bowel obstruction after intestinal surgery. Diagnosis of an internal hernia by imaging findings is not difficult, but preoperative identification of the cause as a transomental hernia is challenging. When patients with an internal hernia are encountered, the decision-making process should be immediately implemented and based on surgical exploration, which is important to prevent further morbidity and mortality. Clinicians should be aware of the possibility of a transomental hernia.

## Conclusions

We experienced a case involving the development of a transomental hernia early after laparoscopic sigmoidectomy. Almost all internal hernias that develop after intestinal surgery are mesenteric hernias; however, we should bear in mind that the more lethal transomental hernia can also rarely occur. Therefore, immediate surgical exploration should be performed for internal hernias, especially for patients with early-onset symptoms after laparoscopic intestinal surgery.

## Data Availability

The dataset supporting the conclusions of this article is available in the Department of Gastrointestinal and Hepato-Biliary-Pancreatic Surgery, Nippon Medical School.

## Abbreviation

CT: Computed tomography
